# Chromosome 15 Imprinting Disorders: Genetic Laboratory Methodology and Approaches

**DOI:** 10.3389/fped.2020.00154

**Published:** 2020-05-12

**Authors:** Merlin G. Butler, Jessica Duis

**Affiliations:** ^1^Division of Research and Genetics, Departments of Psychiatry and Behavioral Sciences and Pediatrics, University of Kansas Medical Center, Kansas City, KS, United States; ^2^Section of Genetics and Inherited Metabolic Diseases, Department of Pediatrics, Children's Hospital Colorado, University of Colorado Anschutz Medical Campus, Aurora, CO, United States

**Keywords:** Prader-Willi syndrome, Angelman syndrome, imprinting disorders, genetic testing flowchart, targeted genetic treatment approaches, duplication 15q, chromosome 15 disorders

## Abstract

Chromosome 15 imprinting disorders include Prader-Willi (PWS) and Angelman (AS) syndromes, which are caused by absent expression from the paternal and maternal alleles in the chromosome 15q11. 2–q13 region, respectively. In addition, chromosome 15q duplication caused by the presence of at least one additional maternally derived copy of the 15q11.2–q13 region can lead to seizures, cognitive and behavioral problems. We focus on PWS and AS in the report, and expand the discussion of clinical care and description with genetic testing to include high-resolution studies to more specifically characterize the molecular mechanisms of disease. The importance of early diagnosis with the necessity for accurate molecular characterization through a step-wise algorithm is emphasized in an era of targeted therapeutic interventions. We present a flowchart to aid in ordering specialized genetic testing as several methods are available for patients presenting with features of PWS and/or AS.

**Graphical Abstract:**
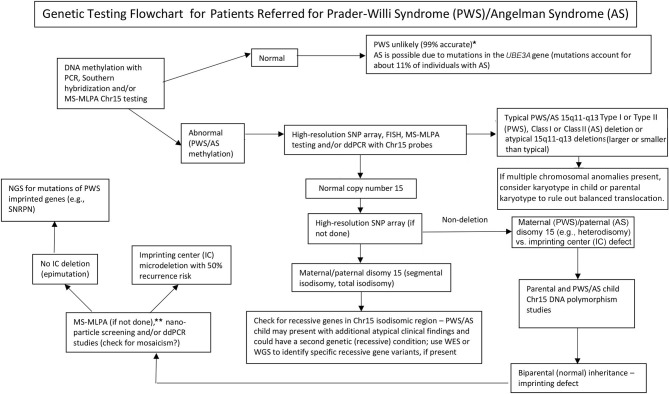
Genetic testing flowchart for patients referred for Prader-Willi syndrome (PWS)/Angelman syndrome (AS). *Rule out Chr15 translocations or inversions by routine chromosome studies; consider other obesity-related genetic disorders; may require fragile X syndrome DNA screening for FMR1 gene repeat expansion or advanced genetic testing with next-generation sequencing (NGS) for FMR1 or other candidate gene variants using whole-exome sequencing (WES) or whole-genome sequencing (WGS; e.g., monogenic causes of obesity). **Can be used to check on methylation status of other Chr15 imprinted genes; ddPCR, droplet digital PCR can be used for mosaicism screening.

## Introduction

The chromosome 15 imprinting disorders include Prader-Willi (PWS) and Angelman (AS) syndromes (1–6) and chromosome 15q duplications. Diagnosis of PWS or AS depends on the parent of origin and whether expression is aberrantly limited to the maternal or the paternal imprinted genes. Duplication 15q is caused by an additional copy of the maternally-derived 15q11.2–q13 region which can lead to seizures, cognitive and behavioral problems including autism spectrum disorder (ASD), but not a PWS or AS phenotype. PWS arises from loss of maternally imprinted and paternally expressed genes from the chromosome 15q11–q13 region, while AS is caused by loss of imprinted and maternally expressed genes in this region, specifically impacting the *UBE3A* gene. Due to the imprinted nature of the responsible genes, both genetic and epigenetic errors can be causative.

In 1989, those with both PWS and non-deletion status were found to have maternal disomy 15 or both 15s from the mother when using polymorphic DNA markers from the proximal 15q11–q13 region (7). Later in the mid-1990s, the development of fluorescent *in situ* hybridization (FISH) DNA probes were used to identify deletions of the 15q11–q13 region in both PWS and AS. Methylation DNA testing was developed during this time period and an abnormal methylation pattern was seen in PWS and AS. Methylation DNA testing is ~99% accurate in identifying the diagnosis of PWS, but will not identify the individual PWS molecular class (2). For AS, DNA methylation testing identifies ~80% of individuals, but again does not distinguish between the molecular classes or detect a mutation in the *UBE3A* gene causing AS.

Microarray technology was developed in the early to mid-2000s and advanced the diagnostic yield. Now the new SNP microarrays include over two million DNA probes and are useful in detecting deletion subtypes and UPD15 subclasses. Other technology such as droplet digital PCR (ddPCR) quantitates copy number using chromosome 15 DNA probes and can diagnose genetic defects in PWS or AS (8). Furthermore, SNP microarrays can identify LOHs defined as >8 Mb in size and when present on chromosome 15 supports the diagnosis of maternal disomy 15 or paternal disomy 15 in the presence of an abnormal DNA methylation pattern for PWS or AS, respectively. Imprinting defect confirmation may require not only SNP microarrays to identify small microdeletions but also parental DNA samples with genotyping to identify the presence of normal (biparental) inheritance of chromosome 15s supporting the presence of an epimutation imprinting defect in PWS or AS thereby impacting recurrence risks. Differentiation of an IC microdeletion from a non-deletion epimutation status is clinically important for families as a 50% recurrence risk is present for additional children if an IC microdeletion is found in the parent (9).

There are over one dozen genes and transcripts in the 15q11–q13 region which appear to play a role in the causation of PWS and/or AS. Genes and transcripts included in the area from the proximal 15q11.2 breakpoints BP1 and the distal 15q13 breakpoint BP3 are *TUBGCP5, CYFIP1, NIPA1, NIPA2, MRKN3, MAGEL2, NDN, NIPAP1, SNURF-SNRPN*, non-coding RNAs (SNORDs), *UBE3A, ATP10A, GABRB3, GABRA5, GABRG3, OCA2*, and *HERC2*. Imprinted *MRKN3, MAGEL2, NDN, NIPAP1*, and *SNURF-SNRPN* genes are paternally expressed and when disturbed may cause features of PWS [e.g., (10)]. For example, *MAGEL2* gene mutations can cause neonatal hypotonia, developmental delay, arthrogryposis, autistic features, a poor suck, and obesity [Schaaf-Yang syndrome; (11)]. Patients have also been reported with features of PWS as a result of small deletions of the non-coding *SNORD116* transcript (12) and other similar deletions in the region (10, 13).

We focused on AS and PWS in this report as both syndromes are detected via DNA methylation testing, which allows for determination of the active parental gene allele and definitive diagnosis in individuals with PWS and in most individuals with AS (2). However, DNA methylation testing will not identify the molecular class in either syndrome. High-resolution chromosome analysis was developed and used in the early 1980s and became a standard laboratory genetic-based test to evaluate for the chromosome 15q11–q13 deletion identified in the majority of patients with PWS at that time (14) and later for AS. The paternal origin of the 15q11–q13 deletion was reported in 1983 (15) and found to be *de novo* but the size of 15q11–q13 deletion or type (typical vs. atypical) could not be determined. Accurate and early diagnosis with identification of the molecular class is essential not only to confirm the clinical diagnosis but also for genetic counseling, to inform care and treatment and to guide expectations. With the intention of ongoing clinical trials, a better understanding of molecular etiology may impact opportunities for patient participation. Furthermore, imminent trials include antisense oligonucleotides to reactivate the silenced paternal copy of the chromosome 15 in individuals with AS.

PWS and AS are complex rare neurodevelopmental disorders due to errors in genomic imprinting. PWS is recognized as the most common genetic cause of life-threatening obesity, if left uncontrolled (2, 4, 6). There are three recognized PWS molecular classes including a paternal 15q11–q13 deletion about 5–6 Mb in size (60% of cases) and maternal disomy 15 (UPD15) in which both chromosome 15s are inherited from the mother (36%) originating from trisomy 15 with loss of the paternal chromosome 15 in early pregnancy leading to two chromosome 15s from the mother (16). The third class is an imprinting center defect. If a microdeletion or epimutation of the imprinting center (IC), which controls the expression status of selected imprinted genes on chromosome 15, is present on the paternal allele then PWS occurs. This imprinting defect is seen in 4% of individuals with PWS (8, 16). Most cases of PWS are sporadic with an approximate equity among ethnic groups and sex. The estimated prevalence of PWS is one in 10,000 to one in 30,000 (2). The number of individuals worldwide with PWS is thought to be ~400,000 with about 20,000 individuals living in the USA (2, 17).

PWS is characterized by infantile hypotonia, a poor suck reflex with feeding difficulties, short stature with small hands and feet, hypogonadism secondary to hormone deficiencies, mild intellectual disability, behavior problems, and hyperphagia often with onset between 6 and 8 years of age that persists into adulthood and results in obesity if environmental controls are not in place. During infancy, characteristic craniofacial features are seen including a narrow bifrontal diameter, strabismus, small upturned nose with a thin upper lip, and down-turned corners of mouth, sticky saliva, and enamel hypoplasia (2, 4, 6, 18). Cognition is generally reduced based on the family background and behavior problems beginning in childhood include self-injury (skin picking), outbursts, stubbornness, and temper tantrums with psychiatric problems occurring during this time or later in adolescence or young adulthood (2). Behavioral problems include anxiety, mood disorders, psychosis, and autism that may correlate with specific PWS genetic subtypes or molecular classes (19).

Historically, PWS is divided into two clinical stages with failure to thrive during infancy representing the first clinical stage and hyperphagia with onset of obesity representing the second stage (2). Later, nutritional phases have been described for this obesity-related genetic disorder and include: Phase 0 with decreased fetal movement and growth retardation in utero, followed by Phase 1 related to hypotonia, failure to thrive with difficulty feeding, Phase 2 beginning at ~2 years of age when weight gain is first noted and Phase 3 when lack of satiety is accompanied by food seeking and hyperphagia leading to obesity, if not externally controlled. Phase 3 begins at around 6–8 years of age (20).

Angelman syndrome is characterized by developmental delay often not apparent until about 6 months of age and subsequent onset of often difficult to control seizures, tremor, wide-based gait, and ataxia with a characteristic happy demeanor (3). There are four recognized molecular mechanisms of AS: *de novo* maternal deletions of chromosome 15q11–q13 (70–80%); mutations of the maternally inherited *UBE3A* gene (10–20%); paternal disomy 15 (3–5%); and imprinting defects (3–5%) within the 15q11–q13 region that alter the expression of the causative *UBE3A* gene (21).

Individuals with AS are often not noticed by medical professionals until ~6 months of age when delays in development in particular delayed motor development are reported. By this time, parents may recognize the happy demeanor that includes frequent laughing, smiling, and excitability. A decreased need for sleep is reported in >80% of individuals with AS (22). They often develop seizures at ages 1–3 years (23). Epilepsy can be intractable and has a characteristic appearance on EEG described as an increased delta power with a characteristic triphasic wave. Individuals with AS are described as ataxic in their movements and walking (24, 25). Microcephaly may develop by ~2 years of age. Stereotypic behaviors include a love of water and crinkly paper and individuals with AS are characteristically non-verbal and categorized as severely intellectual disabled. However, it is notable that individuals with AS have skills not well-captured on the currently available objective neuropsychological tests. They have strong abilities in manipulating electronics, but behaviors can be challenging and include anxiety with short attention spans.

As patients with PWS or AS may present with variable phenotypes depending on the molecular class and because potential treatment and surveillance approaches exist for each, a logical flowchart is needed for ordering genetic tests by the clinician evaluating these patients. The focus of our report is to describe the clinical and genetic findings of these two genomic imprinting disorders and illustrate genetic testing options available in the clinical setting and the order in which the different genetic tests can be obtained most productively.

## Laboratory Genetics Experience in Chromosome 15 Imprinting Disorders

### Prader-Willi Syndrome

To serve as an example of the importance of high-resolution SNP microarray testing, a large multisite cohort of 510 participants with genetically confirmed PWS were recruited in the USA and grouped into three molecular classes. They were further characterized as 15q11–q13 deletion subtypes, maternal disomy 15 subclasses and imprinting center defects (16). In this largest reported PWS cohort, 303 individuals were found to have the 15q11–q13 deletion (60% of cases) composed of 118 individuals (38.9%) having the larger typical 15q11–q13 Type I deletion involving chromosome 15q11–q13 breakpoints BP1 and BP3 and 165 individuals (54.5%) had the smaller typical 15q11–q13 Type II deletion involving breakpoints BP2 and BP3 with 20 individuals having an atypical deletion which is larger or smaller than the typical 15q11–q13 deletion (6.6%). In persons identified to have a deletion of chromosome 15, it is important to consider whether a balanced translocation could be present in the proband's father as this increases the recurrence risk of PWS in the father's offspring. For maternal disomy 15, 185 individuals (36%) had maternal uniparental disomy 15 (UPD15) with 13 individuals (12.5%) having total isodisomy of the entire chromosome 15 due to errors in maternal meiosis II; 60 (57.7%) showed segmental isodisomy from crossover events in maternal meiosis I and 31 showed heterodisomy (29.8%), while 81 individuals did not have SNP microarray analysis and maternal disomy 15 classification determined. Regarding PWS imprinting defects, 22 individuals (4%) were found with 13 (76.5%) having a non-deletion epimutation status, four individuals (23.5%) had a microdeletion of the imprinting center while the remaining five individuals did not have a type of imprinting defect established. In a related study, further analysis of imprinting defects in PWS was carried out by Hartin et al. (8) using droplet digital PCR and next-generation whole-exome sequencing in a separate PWS cohort of 15 unrelated patients and two individuals or 13% were found to have an imprinting center microdeletion defect. In the 60 individuals with segmental isodisomy 15 reported by Butler et al. (16), the total average size of the loss of heterozygosity (LOH) was 25.1 Mb with a range of 5–67.4 Mb and an average size of 16.4 Mb for individual LOHs. Thirty-two individuals had one LOH segment, 25 individuals had two segments and three individuals had three segments. The most common LOH sites were the proximal 15q11–q13 region and distal 15q26 region including the 15q12 and 15q26.1 bands as most commonly recorded.

The presence of maternal UPD15 and specific subclass (segmental or total isodisomy) determination may impact diagnosis and medical care surveillance as a second genetic condition may be present if the mother is a carrier of a recessive gene allele located in the LOH region leading to two identical copies. Hundreds of potentially disease-causing genes are found on chromosome 15 and these diseases should be checked or monitored closely in those with segmental or total isodisomy of chromosome 15. A proposed genetic testing flowchart to identify the different molecular classes for both PWS and AS patients can be seen in Graphical Abstract.

### Angelman Syndrome

Four recognized molecular classes have been identified in AS which may be categorized by the impact on the methylation of the chromosome 15 region. The most common subtype is a deletion of the maternal 15q11.2–q13 region as similarly seen of paternal origin in PWS and found in ~70% of individuals with AS (21). However, in AS the typical Class II deletion is more common. This typical smaller Class II deletion most commonly approximates 5 Mb in size from BP2–BP3 and is present in 50% of deletion AS cases. Class I deletions are 5–7 Mb in size and encompass BP1–BP3 (40% of deletion cases). Atypical deletions may extend from BP1 or BP2–BP4 or more distant breakpoints. In individuals with a deletion on the maternal copy of chromosome 15, one must consider whether there are signs on the chromossomal microarray showing disturbances that indicate there could be a maternal translocation. This increases the recurrence risk of AS in future maternal offspring. Uniparental paternal disomy 15 accounts for 5–7% of individuals with AS. Imprinting defects account for 3–5% of individuals with AS and are caused by defects in the imprinting control center summarized by Buiting et al. (26). In individuals with a defect in the imprint control center, epigenetic marking in the germline fails to properly switch from a paternal pattern with silenced *UBE3A* expression to allow a maternal pattern of expression at the *UBE3A* gene. In as high as 50% of reported cases, a mutation in the imprinting control center may be identified. Mosaic cases of imprinting center defects in which a percentage of cells lack expression of the 15q11.2–q13 region is reported and may be more common than previously thought (27). The final genetic defect in AS does not impact DNA methylation testing results but is caused by a mutation in the maternally inherited *UBE3A* gene. Mutations in this gene account for 11% of AS cases (28). A *UBE3A* mutation could be maternally inherited and therefore it is indicated to do targeted testing in the patient's mother to rule out a 50% recurrence risk in her future offspring. If the mutation is deemed to be inherited, we recommend consideration of testing the patient's maternal grandfather as this could have implications for the maternal aunt's future children.

## Discussion

Medical management of PWS and AS should be directed by a multi-disciplinary team during infancy. Both infants with PWS (more commonly) and AS may have failure to thrive. A dietitian plays an important role in care at first to address failure to thrive and later in childhood to avoid obesity with diet intervention with restriction and use of exercise programs (which is a concern noted more commonly for PWS, but now recognized in AS in some individuals). Clinical geneticists, orthopedic specialists, primary care physicians, specialized occupational (OT), physical (PT) and speech (SLP) therapists, mental health experts, sleep specialists, mental health experts, and endocrinologists are needed to address the multiple health issues in PWS that may occur. An AS team includes clinical geneticists, neurologists, specialized therapists for PT, OT, and SLP services, sleep specialists, gastroenterology, physical medicine and rehabilitation, orthopedics, and mental health experts. For PWS, appropriate medical care, management and counseling are goals to control weight gain and to monitor and treat associated comorbid conditions, behavior, and psychiatric problems. Growth and other hormone deficiencies common in this disorder require treatment. Rigorous control of the diet with food security and a managed routine environment with regular exercise are important strategies to control hyperphagia, obesity and related complications required throughout life. AS requires early intervention including knowledge of specialized therapeutic interventions such as augmentative and assistive communication devices and a strengthening program of intensive developmental exercises and activities for reaching maximal potential (e.g., SPIDER), early treatment with benzodiazepines for seizures and diet therapy such as use of a ketogenic diet. Maximizing all aspects of care including sleep disorders and constipation greatly influence seizure control. A specialized center familiar with the intricacies and unique aspects of these disorders can affect outcome.

Early diagnosis is vital to ensure early intervention for both PWS and AS. For PWS, an early diagnosis should be made during infancy to initiate growth hormone treatment, manage feeding concerns, obesity, hormone deficiencies, developmental delays, and behavioral problems. Diagnosis in AS also ensures early therapies which impact developmental outcomes, as well as seizure prophylaxis including preparation with appropriate benzodiazepines. Other interventions that may prove beneficial include specialized diets for individuals with AS such as the ketogenic diet or low glycemic index therapy (LGIT). Early diagnosis may also lower the costs of medical care by preventing extended hospitalizations related to feeding problems in individuals with PWS and seizures for children with AS.

Identifying the PWS or AS molecular class with advanced genetic testing such as high-resolution SNP microarrays will allow more accurate diagnosis, leading to better indicators for prognosis, and more accurate genetic counseling of family members. High-resolution SNP microarrays, FISH analysis, methylation specific-multiplex ligation probe amplification (MS–MLPA), and/or chromosome 15 genotyping are all useful in determining 15q11–q13 deletions. High-resolution SNP microarrays can identify the deletion subtypes (typical and atypical) in both PWS and AS, and UPD15 subclasses (segmental isodisomy, and total isodisomy). The heterodisomy subtype of UPD and IC defects (microdeletion and epimutation) in both PWS and AS may require additional diagnostic work up as illustrated in Graphical Abstract. The subtype or classes impacts diagnosis, potential recurrence risk for family members, prognosis and monitoring for other genetic conditions and high-risk features related to the molecular class. For example, autistic features and psychosis are more common in those with PWS and maternal disomy 15 and may relate to the specific UPD15 subclasses. Those with the larger Class I deletions in AS are more likely to develop difficult to treat seizures and microcephaly.

A genetic testing flowchart incorporating testing options that are available including those used historically for both PWS and AS are listed in Graphical Abstract. Testing for PWS or AS often begins with DNA methylation and if abnormal then advances to other genetic testing methods including high-resolution SNP microarrays or MS-MLPA assays based on availability to the clinicians and families in their clinical setting. Preferably, a high-resolution SNP array would be ordered which is readily and commercially available in Westernized medical care. Next-generation sequencing (NGS) of the exome (or whole genome) is also available for clinicians but droplet digital PCR (ddPCR) is currently research-based (14). SNP arrays can identify specific molecular classes in the majority of patients presenting with features of PWS (about 85% of cases) or AS (about 80%) while the remaining patients will need additional testing as described in Graphical Abstract. Specific advanced genetic testing (e.g., ddPCR) may be appropriately sensitive to quantify mosaicism and may identify a diagnosis in a large subset of individuals with milder clinical features of PWS and AS, but more research is needed.

Early clinical differences were found when comparing those with PWS or AS having the deletion vs. non-deletion status (29) including hypopigmentation in those with PWS and AS having the 15q11–q13 deletion (30). Later, higher verbal IQ scores (31) or psychosis (32) were reported in those with maternal UPD15 compared to deletion in individuals with PWS. Furthermore, Butler et al. (19) reported lower adaptive scores and more obsessive-compulsive behaviors in PWS individuals with the 15q11–q13 Type I deletion compared with UPD15. Zarcone et al. (33) reported individuals with PWS and the 15q11–q13 Type I deletion had more compulsions with personal cleanliness and compulsive behavior that was difficult to interrupt and interfered with social activities more so than those with Type II deletions or UPD15. In a phenotype-genotype correlation study in Angelman syndrome, Moncla et al. (34) reported increased seizure activity in those with the larger Class I deletion compared with non-deletion. Microcephaly, ataxia, hypotonia, and feeding difficulties are also more likely in the deletion subtype (3). They may have more severe language impairment in particular receptive language and autistic traits (21, 35). Individuals with AS with paternal UPD may have improved receptive language, improved motor abilities, and a decreased prevalence of seizures. Mosaic individuals may also have a milder phenotype including improved language abilities, adaptive functioning, and fewer seizures (36).

Next-generation exome or whole-genome sequencing may also have a place in genetic evaluations in PWS or AS, particularly in those individuals presenting with unusual findings or delayed diagnosis (e.g., UPD15 segmental or total isodisomy) and in cases where parental DNA is not available (8). To address the use and type of genetic testing for PWS and AS, a new genetic testing flowchart was developed for the clinician as described and illustrated in Graphical Abstract. This flowchart can assist in ordering genetic testing based on clinical presentation to determine appropriate diagnosis, management, and treatment and to supply the most accurate genetic counseling information for other family members. We suggest the use of this algorithm to definitively complete the diagnostic work up for both PWS and AS. We argue the diagnosis is incomplete without knowledge of the patient's specific genetic subtype to guide counseling, anticipatory guidance, management and likely therapeutic options. The molecular class determination is important for medical care and treatment and helpful for the clinician engaged in genetic counseling of family members for PWS or AS.

## Author Contributions

MB and JD contributed to drafting the manuscript, review of literature, contributed their expertise and edited the manuscript.

## Conflict of Interest

The authors declare that the research was conducted in the absence of any commercial or financial relationships that could be construed as a potential conflict of interest.

## References

[1] Bittel DC Butler MG Prader-Willi syndrome: clinical genetics, cytogenetics and molecular biology. Expert Rev Mol Med. (2005) 25:1–20. 10.1017/S1462399405009531 PMC6750281 16038620

[2] Butler MG Lee PDK Whitman BY. Management of Prader-Willi Syndrome. New York, NY: Springer. (2006). 10.1007/978-0-387-33536-0

[3] Williams CA Driscoll DJ Dagli AI. Clinical and genetic aspects of Angelman syndrome. Genet Med. (2010) 12:385–95. 10.1097/GIM.0b013e3181def138 20445456

[4] Cassidy SB Schwartz S Miller JL Driscol DJ. Prader-Willi syndrome. Genet Med. (2012) 14:10–26. 10.1038/gim.0b013e31822bead0 22237428

[5] Angulo MA Butler MG Cataletto ME. Prader-Willi syndrome: a review of clinical, genetic, and endocrine findings. J Endocrinol Invest. (2015) 38:1249–63. 10.1007/s40618-015-0312-9 26062517 PMC4630255

[6] Butler MG. Single gene and syndromic causes of obesity: illustrative examples. Prog Mol Biol Transl Sci. (2016) 140:1–45. 10.1016/bs.pmbts.2015.12.003 27288824 PMC7377403

[7] Nicholls RD Knoll JH Butler MG Karam S Lalande M. Genetic imprinting suggested by maternal heterodisomy in nondeletion Prader-Willi syndrome. Nature. (1989) 342:281–5. 10.1038/342281a0 2812027 PMC6706849

[8] Hartin SN Hossain WA Francis D Godler DE Barkataki S Butler MG. Analysis of the Prader-Willi syndrome imprinting center using droplet digital PCR and next-generation whole-exome sequencing. Mol Genet Genomic Med. (2019) 7:e00575. 10.1002/mgg3.575 30793526 PMC6465664

[9] Hartin S Hossain WA Weisensel N Butler MG. Three siblings with Prader-Willi syndrome caused by imprinting center microdeletions and review. Am J Med Genet A. (2018) 176:886–95. 10.1002/ajmg.a.38627 29437285 PMC6688622

[10] Hassan M Butler MG. Prader-Willi syndrome and atypical submicroscopic 15q11-q13 deletions with or without imprinting defects. Eur J Med Genet. (2016) 59:584–9. 10.1016/j.ejmg.2016.09.017 27659713 PMC6688621

[11] Fountain MD Schaaf CP Prader-Willi syndrome and Schaaf-Yang syndrome: neurodevelopmental diseases intersecting at the MAGEL2 gene. Diseases. (2016) 13:4 10.3390/diseases4010002 PMC5456300 28933382

[12] Sahoo T del Gaudio D German JR Shinawi M Peters SU Person RE. Prader-Willi phenotype caused by paternal deficiency for the HBII-85 C/D box small nucleolar RNA cluster. Nat Genet. (2008) 40:719–21. 10.1038/ng.158 18500341 PMC2705197

[13] Tan Q Potter KJ Burnett LC Orsso CE Inman M Rhyman DC Prader-Willi-Like phenotype caused by an atypical 15q11.2 microdeletion. Genes (Basel). (2020) 25:11 10.3390/genes11020128 PMC7073628 31991769

[14] Ledbetter DH Riccardi VM Airhart SD Strobel RJ Keenan BS Crawfrd JD. Deletions of chromosome 15 as a cause of the Prader-Willi syndrome. N Engl J Med. (1981) 304:325–9. 10.1056/NEJM198102053040604 7442771

[15] Butler MG Palmer CG Parental origin of chromosome 15 deletion in Prader-Willi syndrome. Lancet. (1983) 4:1285–6. 10.1016/S0140-6736(83)92745-9 PMC5510872 6134086

[16] Butler MG Hartin SN Hossain WA Manzardo AM Kimonis V Dykens E. Molecular genetic classification in Prader-Willi syndrome: a multisite cohort study. J Med Genet. (2019) 56:149–53. 10.1136/jmedgenet-2018-105301 29730598 PMC7387113

[17] Butler MG Thompson T Prader-Willi syndrome: clinical and genetic findings. Endocrinologist. (2000) 10:3s−16s. 10.1097/00019616-200010041-00002 27570435 PMC4996620

[18] Butler MG. Prader-Willi syndrome: current understanding of cause and diagnosis. Am J Med Genet. (1990) 35:319–32. 10.1002/ajmg.1320350306 2309779 PMC5493042

[19] Butler MG Bittel DC Kibiryeva N Talebizadeh Z Thompson T. Behavioral differences among subjects with Prader-Willi syndrome and type I or type II deletion and maternal disomy. Pediatrics. (2004) 113:565–73. 10.1542/peds.113.3.565 14993551 PMC6743499

[20] Miller JL Lynn CH Driscoll DC Goldstone AP Gold JA Kimonis V Nutritional phases in Prader-Willi syndrome. Am J Med Genet A. (2011) 155:1040–9. 10.1002/ajmg.a.33951 PMC3285445 21465655

[21] Lossie AC Whitney MM Amidon D Dong HJ Chen P Theriaque D. Distinct phenotypes distinguish the molecular classes of Angelman syndrome. J Med Genet. (2001) 38:834–45. 10.1136/jmg.38.12.834 11748306 PMC1734773

[22] Trickett J Oliver C Heald M Denyer H Surtess A Clarkson E. Multi-method assessment of sleep in children with Angelman syndrome: a case-controlled study. Front Psychiatry. (2019) 10:874. 10.3389/fpsyt.2019.00874 31849727 PMC6895248

[23] Buiting K Clayton-Smith J Driscoll DJ Gillessen-Kaesback G Kanber D Schwinger E. Clinical utility gene card for: Angleman syndrome. Eur J Hum Genet. (2015) 23:2. 10.1038/ejhg.2014.93 24896151 PMC4297916

[24] Pelc K Cheron G Dan B. Behavior and neuropsychiatric manifestations in Angelman syndrome. Neuropsychiatr Dis Treat. (2008) 4:577–84. 10.2147/NDT.S2749 18830393 PMC2526368

[25] Bindels-de Heus KGCB Mous SE Hooven-Radstaake MT van Iperen-Kolk B Navis C Rietman AB. An overview of health issues and development in a large clinical cohort of children with Angelman syndrome. Am J Med Genet A. (2020) 182:53–63. 10.1002/ajmg.a.61382 31729827 PMC6916553

[26] Buiting K Williams C Horsthemke B. Angelman syndrome - insights into a rare neurogenetic disorder. Nat Rev Neurol. (2016) 12:584–93. 10.1038/nrneurol.2016.133 27615419

[27] Le Fevre A Beygo J Silveira C Kamien B Clayton-Smith J Colley A. Atypical Angelman syndrome due to a mosaic imprinting defect: case reports and review of the literature. Am J Med Genet A. (2017) 173:753–7. 10.1002/ajmg.a.38072 28211971

[28] Margolis SS Sell GL Zbinden MA Bird LM. Angelman syndrome. Neurotherapeutics. (2015) 12:641–50. 10.1007/s13311-015-0361-y 26040994 PMC4489961

[29] Butler MG Meaney FJ Palmer CG. Clinical and cytogenic survey of 39 individuals with Prader-Labhart-Willi syndrome. Am J Med Genet. (1986) 23:793–809. 10.1002/ajmg.1320230307 3953677 PMC5494992

[30] Butler MG. Hypopigmentation: a common feature of Prader-Labhart-Willi syndrome. Am J Hum Genet. (1989) 45:140–146. 2741944 PMC1683374

[31] Roof E Stone W MacLean L Feurer ID Thompson T Butler MG. Intellectucal characteristics of Prader-Willi syndrome: comparison of genetic subtypes. J Intellect Disabil Res. (2000) 44(Pt 1):25–30. 10.1046/j.1365-2788.2000.00250.x 10711647 PMC6790137

[32] Boer H Holland A Whittington J Butler J Webb T Clarke D. Psychotic illness in people with Prader-Willi syndrome due to chromosome 15 maternal uniparental disomy. Lancet. (2002) 359:135–6. 10.1016/S0140-6736(02)07340-3 11809260

[33] Zarcone J Napolitano D Peterson C Breidbord J Ferraioli S Caruso-Anderson M. The relationship between compulsive behavior and academic achievement across the three genetic subtypes of Prader-Willi syndrome. J Intellect Disabil Res. (2007) 51:478–87. 10.1111/j.1365-2788.2006.00916.x 17493030 PMC6706850

[34] Moncla A Malzac P Voelckel MA Auquier P Girardot L Mattei MG. Phenotype-genotype correlation in 20 deletion and 20 non-deletion Angelman syndrome patients. Eur J Hum Genet. (1999) 7:131–9. 10.1038/sj.ejhg.5200258 10196695

[35] Sahoo T Bacino CA German JR Shaw CA Bird LM Kimonis V. Identification of novel deletions of 15q11q13 in Angelman syndrome by array-CGH: molecular characterization and genotype-phenotype correlations. Eur J Hum Genet. (2007) 15:943–9. 10.1038/sj.ejhg.5201859 17522620

[36] Carson RP Bird L Childers AK Wheeler F Duis J Preserved expressive language as a phenotypic determinant of mosaic Angelman syndrome. Mol Genet Genomic Med. (2019) 1:e837 10.1002/mgg3.837 PMC6732290 31400086

